# Wet Granulation as a Solidification Strategy for Converting Aqueous Nanosuspensions into Solid Dosage Forms

**DOI:** 10.3390/pharmaceutics18050543

**Published:** 2026-04-28

**Authors:** Erasmo Ragucci, Marco Uboldi, Alice Melocchi, Mauro Serratoni, Lucia Zema

**Affiliations:** 1 PhormulaMi Research Group, Sezione di Tecnologia e Legislazione Farmaceutiche “Maria Edvige Sangalli”, Dipartimento di Scienze Farmaceutiche, Università degli Studi di Milano, via Giuseppe Colombo 71, 20133 Milano, Italy; 2 Novartis Pharma AG, 4056 Basel, Switzerland

**Keywords:** BCS class II drugs, water-based nanosuspensions, wet granulation, solid dosage forms, mini-tablets, immediate release

## Abstract

**Background:** The well-established biopharmaceutical advantages provided by nanosuspensions (NSs) make their conversion into solid oral dosage forms particularly appealing for improving both patients’ compliance and product stability. However, such a transformation continues to represent a significant challenge. **Aim:** This study explored manual wet granulation (WG) as a laboratory-scale strategy to transform an NS containing cinnarizine (CN) into granules, which were intended for direct administration or to be further processed. **Methods:** A range of polymers, characterized by different interaction mechanisms with aqueous fluids, were employed as carriers to incorporate the CN-containing NS during manual WG. The resulting granules were thoroughly characterized before tableting. **Results:** The NS-loaded products exhibited satisfactory physio-technological properties, effective nanocrystal redispersibility, high drug load efficiency, and expected in vitro performance. Moreover, they turned out to be suitable intermediates for mini-tablet production. **Conclusions:** Based on the data collected, WG turned out to be an effective lab-scale method for transforming an aqueous CN-containing NS into solid products (i.e., granules and mini-tablets), while preserving the starting properties of the drug nanocrystals. By adjusting formulation and process parameters, a variety of release kinetics were achieved, highlighting the value of the pursued approach, especially for early-stage screening of new drug candidates belonging to class II of the biopharmaceutical classification system.

## 1. Introduction

In the drug delivery field, the increasing awareness of the advantages associated with the application of nanotechnologies has driven substantial progress in the formulation of poorly soluble drugs [1,2]. In this context, the development of nanosuspensions (NSs) has emerged as a powerful tool to enhance the dissolution rate and improve the oral bioavailability of challenging molecules [3,4]. However, as water-based systems, NSs present several limitations: they are often characterized by restricted long-term stability, are difficult to handle from the patients’ perspective, which could in principle compromise compliance, and pose major challenges in terms of final product storage and distribution [5,6,7,8,9]. Therefore, developing robust strategies to transform liquid NSs into solid dosage forms has become a key research priority, as relevant solidification can markedly improve stability, streamline the supply chain, and ultimately maximize both the therapeutic and the commercial potential of nanosized drugs [4,10,11,12]. In addition, the availability of NS-containing solid dosage forms, even during the early phases of pharmaceutical research, can be highly advantageous, in principle accelerating formulation screening and reducing overall time and cost of development.

Several solidification approaches have been tested, especially at the research level, to convert NSs into solid dosage forms, each characterized by peculiar advantages and limitations [4,13]. By way of example, spray drying enables continuous, scalable production of dry powders and offers good control over particle morphology and size but requires careful thermal and formulation control to avoid nanocrystal aggregation and degradation [14,15]. For drugs sensitive to temperature, freeze drying is the process of choice, but it is known for being time-consuming and expensive, leading to highly porous powders that may require further processing [16,17]. More recently, hot melt extrusion has also been explored, being a solvent-free, continuous process able to embed NSs into diverse polymeric carriers, but its use is limited by the active ingredient thermal stability [18,19]. On the other hand, wet granulation (WG) has been described as an advantageous alternative for converting NSs into solid products if the NS itself is used as the granulating liquid [4,20,21]. Indeed, WG transforms fine powders into larger, uniform granules by promoting inter-particulate adhesion, generating a cohesive wet mass that, after proper drying and screening, yields granules with improved flowability, bulk density, and mechanical strength [22,23,24,25]. From a processing perspective, WG involves multiple steps, named wetting, nucleation, coalescence-driven growth, consolidation, and attrition, during which formulation variables and process parameters would critically affect the final granule structure. Key granule attributes, and especially the particle size, morphology, moisture content, and hardness, are fundamental to final product quality, whether granules are intended for direct administration or used as intermediates for downstream processing [26,27,28,29].

Industrial WG can be implemented in several automated formats [29,30]. High-shear wet granulation affords tight control of shear and liquid addition for reproducible granule size and density, while twin-screw wet granulation offers a continuous, scalable alternative with improved residence-time control and reduced solvent consumption [31,32,33,34,35]. Finally, fluid-bed granulation integrates spraying and drying in a single step, leading to a relatively high-porous granule with improved compaction attitude [36,37,38]. These automated methods generally improve reproducibility and yield compared with manual approaches, but they require greater capital investment and material throughput for process optimization [29]. On the other hand, manual WG represents an accessible solidification strategy that is particularly valuable during early-stage formulation screening [30,39]. In contrast to equipment-intensive technologies, this process requires minimal infrastructure and energy input, while enabling rapid iterative testing, particularly when only limited quantities of drug or excipients are available. This makes it a low-cost and low-risk platform for preliminary compatibility and feasibility studies, allowing early identification of promising formulation strategies before transitioning to semi-automated or fully automated granulation systems. Indeed, it provides direct visual and tactile control over liquid addition, wetting behavior, and granule growth kinetics, process attributes that are hard to check in closed automated systems. This hands-on method would support better understanding of nucleation, coalescence, and consolidation phenomena, which are fundamental for promoting rational formulation design [40]. This technique allows rapid adjustment of process and formulation variables without the constraints imposed by automated control systems, supporting hypothesis-driven experimentation during early development phases. However, manual WG also encompasses several limitations. It is inherently operator-dependent, and variability in mixing intensity, liquid distribution, and endpoint determination can lead to limited reproducibility in granule size distribution and mechanical properties, resulting in batch-to-batch inconsistency [30]. Furthermore, manual WG lacks objective, real-time monitoring tools that are increasingly available in automated systems, reducing the ability to precisely control endpoint parameters such as granule density, porosity, and liquid saturation. Nevertheless, the combination of accessibility, flexibility, and the possibility to gather direct insights into the granule formation makes manual WG a valuable complementary approach to automated technologies, particularly in the early phases of formulation development where rapid, low-resource and high-throughput screening is essential.

Based on these premises, the present work investigated, at the lab scale, the feasibility of using WG to convert a water-based NS into granules. In more detail, granules were produced using a cinnarizine-containing NS (CN NS) as the granulation liquid, which served as a case study. In fact, CN was chosen as a representative compound of class II Biopharmaceutical Classification System (BCS), a category that encompasses a large proportion of new chemical entities (NCEs) [41]. Indeed, cinnarizine exhibits extremely low aqueous solubility and pH-dependent dissolution, making it an interesting benchmark for assessing the impact of particle size reduction on dissolution enhancement [42,43]. Moreover, CN has a favorable safety profile, which allows its employment at relatively high concentrations in formulation studies without raising significant safety concerns for the operators [44,45]. These combined features make it an excellent model compound for the present study. To maximize versatility, polymeric excipients showing a variety of interaction mechanisms with aqueous fluids, thus potentially enabling a range of release profiles from immediate to modified release, were investigated as carriers. On the other hand, povidone and copolymer of vinylpyrrolidone and vinyl acetate were tested as binders in view of their good aqueous solubility and because they have been reported to promote granule nucleation, reduce the fraction of fines, and improve granule mechanical strength, while offering a range of wetting and rheological behaviors [46]. The resulting granules were assessed for key quality attributes considering their potential use either as final dosage forms or as intermediates for subsequent tablet manufacturing.

## 2. Materials and Methods

### 2.1. Materials

CN (mean particle size distribution, PSD: ~63.5 ± 1.2 μm) and CN NS (CN load, 10 wt%; d-a-tocopheryl polyethylene glycol 1000 succinate, TPGS, 2.5%; Z-average of ~ 350 nm and PdI of 0.4) were both kindly supplied by Novartis Pharma AG (Basel, Switzerland). Specifically, CN NS was manufactured and characterized following an experimental protocol previously validated within the company [18,47]. Sodium starch glycolate (Explotab^®^ CLV, EXP; JRS PHARMA, Rosenber, Germany); soy polysaccharides (Emcosoy^®^, SP; JRS PHARMA, Rosenber, Germany); lactose monohydrate (GranuLac^®^ 200, LT; MEGGLE, Wasserburg, Germany); polymethacrylates (Eudragit^®^ RL PO, ERL; Eudragit^®^ E PO, EPO; Evonik, Essen, Germany); ethylcellulose (Ethocel™ standard 10 premium, EC; Dupont, UK); hydroxypropyl methylcellulose acetate succinate (AQQAT^®^ AS LG, HPMCAS; Shin-Etsu Chemical, Tokyo, Japan). Binders: polyvinylpyrrolidone (Kollidon^®^ 30, PVP K-30; BASF, Mannheim, Germany); copolymer of vinylpyrrolidone and vinyl acetate 6:4 (Kollidon^®^ VA 64, PVP/VA 64; BASF, Germany). Other additives: potassium dihydrogen phosphate (AnalaR NORMAPUR; VWR CHEMICALS, Leuven, Belgium); methanol (AnalaR NORMAPUR, VWR CHEMICALS, Fontenay-sous-Bois, France); hydroxypropyl methylcellulose (METHOCEL™ K4M premium, HPMC K4M; Colorcon, Harleysville, PA, USA), magnesium stearate (Mg St; Sigma-Aldrich, Milan, Italy).

### 2.2. Methods

#### 2.2.1. CN NS Characterization

Particle size (Z-average, Z-avg) and polydispersity (PdI) of CN NS (n = 3) were measured using Zetasizer Nano ZS Dynamic Light Scattering (DLS) instrument (Malvern Instruments, Worcestershire, UK). Before the analysis, samples were diluted (1:100) using a saturated solution of CN. The resulting dispersions were sonicated (Ultrasonic Homogenizer UP200St, Hielscher, Teltow, Germany) for 1 min at 70% amplitude (corresponding to approximately 4–6 kJ) to disrupt potential aggregates and ensure proper dispersion of the nanocrystals. Then, an aliquot of the sample solution was withdrawn and equilibrated for 120 s to ensure proper conditioning. The temperature was set at 25 °C, and each analysis involved 10 measurements (100 s duration each). The stability of the CN NS, stored in a refrigerator (VWR, Basel, Switzerland; 3 ± 1 °C), was checked by measuring the Z-avg and PdI at pre-established time periods (i.e., up to 18 months).

#### 2.2.2. WG

WG was performed manually using a mortar and pestle. Composition of the various formulations tested and relevant codes are reported in Table 1. Granules were prepared using either aqueous binder solutions or the CN NS supplemented with binders as the granulating liquid. Specifically, two different binder types (i.e., PVP K-30 and PVP VA/64) at two increasing concentrations (i.e., 2.5 wt% and 5 wt%) were tested. The required amount of binder was accurately weighed (analytical balance, EWJ 600-2SM, KERN & SOHN GmbH, Balingen, Germany) directly into the liquid phase (i.e., either water or CN NS) and stirred until complete dissolution before use.

For all batches, the same quantity of granulation liquid was added gradually in small aliquots, while manually mixing the powder blend with a pestle to ensure uniform wetting and binder distribution. This procedure was applied to both placebo (i.e., carrier granulated with water-based binder solutions) and drug-containing (i.e., carrier granulated with CN NS supplemented with binders) formulations in order to achieve comparable wetting conditions and binder content. Liquid addition was interrupted once target conditions were reached, which meant, for NS-based batches, when the desired CN loading (i.e., 100 mg/g) was achieved, while for placebo batches, when an equivalent liquid quantity had been incorporated to match the wetting and binder content of the corresponding NS-based formulations. After the wet mass was broken down into granules by passing it through a 1000 µm sieve, the resulting material was oven-dried at 40 °C for 8 h until a constant weight was achieved.

Granules with a particle size between 355 and 500 µm were selected using a vibratory sieve shaker (AS 200 basic, Retsch, Haan, Germany, time = 4 min, and amplitude of sieving = 5). The product yield was calculated as the percentage ratio between the mass of granulate retained on the 355 µm sieve and the total mass of granules produced, as reported below:

(1) $$\mathrm{Yield}\;\left(\%\right)\;=\;100\;\times \;\frac{\mathrm{weight}\;\mathrm{of}\;\mathrm{granules}\;(\mathrm{size}\;\mathrm{fraction}\;500–355\;µm)}{\mathrm{total}\;\mathrm{weight}\;\mathrm{of}\;\mathrm{granules}\;\mathrm{produced}\;}$$

This intermediate size range was chosen because it typically provides the best compromise between minimizing fines (which are known to impair flow, increase dusting and segregation) and avoiding oversized granules that can compromise further processing [28,48].

#### 2.2.3. Granules Characterization

##### 2.2.3.1. Density

A total of 5 g of granules within the 355–500 µm size range were accurately weighed (analytical balance, EWJ 600-2SM, KERN & SOHN GmbH, Balingen, Germany) and transferred into a 25 mL graduated cylinder. The apparent volume (V_0_) was recorded, and the bulk density ($\rho_{b}$) was calculated as follows:

(2) $$\rho_{b}\;(g/\mathrm{mL})\;=\;\frac{m}{V_{0}}$$

Tapped density ($\rho_{t}$) was then determined according to Method 1 of the European Pharmacopoeia (Eur. Ph. XI Ed., 2.9.34). The 25 mL cylinder containing the granules was placed in a tapping volumeter (Stampf Volumeter Stav2003, JEL, Gemini, Amsterdam, The Netherlands). After 250 taps, the final volume (V_f_, corresponding to V_250_) was recorded and $\rho_{t}$ was calculated as follows:

(3) $$\rho_{t}\;(g/\mathrm{mL})\;=\;\frac{m}{V_{f}}$$

##### 2.2.3.2. Flowability

Granule flowability (Eur. Ph. XI Ed., 2.9.36) was assessed by calculating the compressibility index (CI), according to the following equations:

(4) $$\mathrm{CI}\;(\%)\;=\;100\;\times \;\frac{V_{0}-V_{f}}{V_{0}}$$

##### 2.2.3.3. Friability

Friability of granules within the 355–500 µm size range was evaluated using a procedure adapted from the European Pharmacopoeia (Eur. Ph. XI Ed., 2.9.7, Method B). In particular, 4 g of granules were weighed (analytical balance, EWJ 600-2SM, KERN & SOHN GmbH, Balingen, Germany) directly into a glass container, which was then sealed. The container was placed in a turbula mixer (Turbula T2C, Willy A. Bachofen WAB, Muttenz, Switzerland) and subjected to horizontal oscillations for 2 min at a constant frequency of 200 oscillations/min. After treatment, the granules were sieved using a pre-calibrated set of sieves (i.e., 500 µm, 355 µm, and collecting base), mounted on a vibratory sieve shaker (AS 200 basic, Retsch, Germany). Sieving was performed for 1 min at an amplitude of 4. The fraction retained on the 355 µm sieve was collected and weighed (CKE 16K0.05, KERN & SOHN, Germany). Friability was calculated as mass loss, according to the following equation:

(5) $$\mathrm{Mass}\;\mathrm{loss}\;(\%)\;=\;100\;\times \;\frac{m_{1}-m_{2}}{m_{1}}$$

where m_1_ is the initial mass of granules, and m_2_ is the final mass after sieving.

##### 2.2.3.4. Particle Size

Particle size analysis (n = 3) of the selected granulates was carried out using a laser diffraction particle size analyzer (Mastersizer 3000, Malvern Instrument, Malvern, UK) equipped with a dry powder disperser (Aero S). The mean particle size (D_50_) and the corresponding standard deviation (SD) were subsequently calculated.

##### 2.2.3.5. Drug Content

CN content (n = 3) was quantified using high-performance liquid chromatography (HPLC; HP 1100 ChemStations, Agilent Technologies, Milan, Italy) equipped with an UV-Vis detector and a reverse-phase column L-column (InertClone ODS, 150 mm × 4.6 mm, 5 μm ODS (3) 100 Å, Phenomenex Inc., Aschaffenburg, Germany), maintained at 30 °C. The mobile phase consisted of methanol and pH 3.5 phosphate buffer (70:30 *v*/*v*), filtered through a 0.45 µm membrane prior to use, and delivered at a flow rate of 1 mL/min. The run time was set to 10 min. For the analysis, samples (25 mg) were dispersed in 15 mL of mobile phase and then ultrasonicated at 25 °C for 30 min to ensure complete CN dissolution. The resulting liquid was filtered through a 0.22 μm syringe filter and transferred into an HPLC vial kept at 30 °C. A 10 μL aliquot was automatically injected from the vial into the HPLC system for spectrophotometric detection (retention time = 4.7 min, λ = 250 nm). The CN content was calculated using a purposely-built calibration curve in the 0.25–500 µg/mL range (y = 33.581x + 5.0951, R^2^ = 0.9998). The percentage CN content was calculated as the ratio between the experimentally measured drug load and a normalized reference value, the latter being calculated taking into account the actual drug concentration in the CN NS (92.62 µg/mL).

##### 2.2.3.6. In Vitro Performance

Dissolution performance (n = 3, samples of 25 ± 2 mg in weight) was evaluated in HCl 0.1N (pH 1.2) using a USP Apparatus 4 (Sotax, Allschwil, Switzerland) equipped with powder/granule cells. The apparatus was operated in the open-loop mode, with a continuous flow of fresh media into the cells (8 mL/min), and automatic sampling of the latter at pre-established time points (5 mL every 5 min). CN content in the sample was quantified by HPLC, as described in Section 2.2.3.5. Results were expressed as a percentage of the CN released with respect to the actual drug content in the tested product. HCl 0.1 N was employed as the dissolution medium based on both biopharmaceutical and analytical considerations [42,49,50]. CN exhibits high solubility in acidic environments, ensuring sink conditions throughout the test and preventing solubility-related artefacts that could obscure differences in release kinetics. This way, the dissolution method setup was capable of detecting subtle formulation-dependent differences that might appear superimposable in different media. For this reason, the use of acidic media has been widely reported in the scientific literature for the in vitro evaluation of CN-containing products.

##### 2.2.3.7. Nanocrystals Redispersibility

Nanocrystals redispersibility (n = 3) was measured by DLS, using a Zetasizer Nano ZS DLS instrument (Malvern Instruments, Worcestershire, UK). Samples were dispersed in a saturated CN solution under magnetic stirring (200 rpm), and the resulting dispersions were sonicated (Ultrasonic Homogenizer UP200St, Hielscher, Germany) for 1 min at 70% amplitude (corresponding to approximately 4–6 kJ) to separate finer excipient particles from the drug nanocrystals. Prior to the dimensional analysis, the insoluble excipient particles were allowed to sediment, and the supernatant was carefully collected for analysis. Then, samples were equilibrated for 120 s to ensure proper conditioning. The temperature was set at 25 °C, and each analysis involved 10 measurements (100 s duration each). Z-avg and PdI were used as characteristic parameters for differentiating the granules in terms of capability of redispersing the CN nanocrystals. Data were compared to those relevant to the original NS dimension through the RDI [51]. More in detail, RDI values were calculated (Equation (6)) by normalizing the mean particle size of the redispersed nanocrystals (Z-avg. redisp) to the mean particle size of the original NS (Z-avg.orig):

(6) $$\mathrm{RDI}\;=\;\frac{Z\text{-}\mathrm{avg}.\mathrm{redisp}}{Z\text{-}\mathrm{avg}.\mathrm{orig}}$$

#### 2.2.4. Tableting

**Figure 1:**
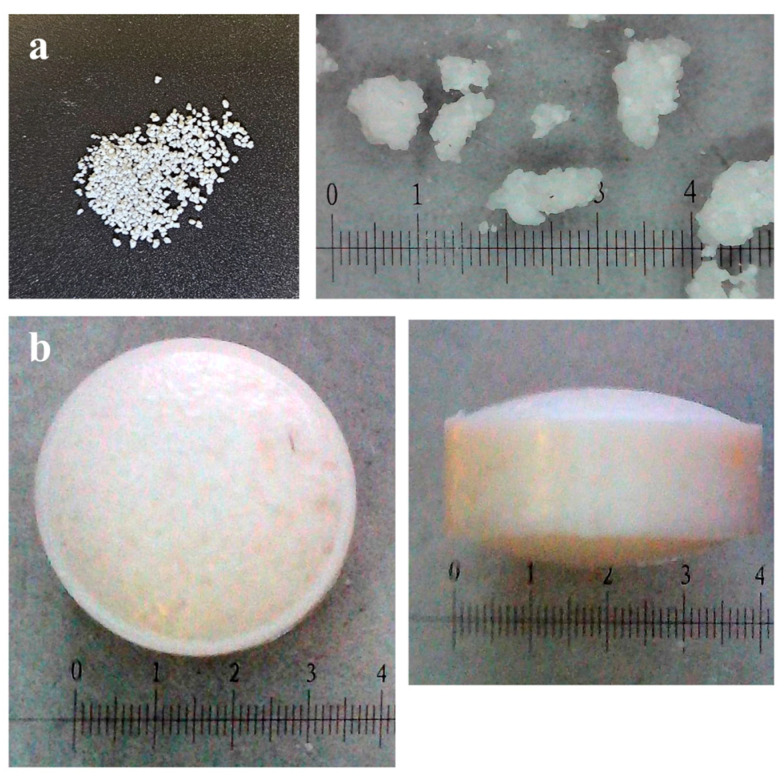
Photographs of EC-based (**a**) granules and (**b**) mini-tablets.

Tablets with a nominal weight of 25 mg were prepared based on the EC2bNS formulation (Figure 1). Granules (355–500 µm particle size) were blended with an external phase consisting of HPMC K4M and/or Mg St, as detailed in Table 2. The resulting blends were compressed by means of a rotary tablet press (AM-8S, Officine Ronchi, Cinisello Balsamo, Italy) equipped with 4 mm round punches.

#### 2.2.5. Mini-Tablet Characterization

Mini-tablets (n = 10) were characterized for mass (CRYSTAL 500, Gibertini, Milan, Italy), thickness (Mitutoyo CD-15CP, Andover, UK), and hardness (crash tester, Erweka GmbH, Langen, Germany). Drug load and dissolution performance were evaluated as discussed in Section 2.2.3.5 and Section 2.2.3.6, respectively. However, for the in vitro performance, the time points differed from those previously used when dealing with granules and were set at 15, 30, 60, 90, and 120 min.

## 3. Results

### 3.1. Binders and Carriers Selection

In this feasibility study, WG was selected as a relatively easy and low-cost strategy for converting water-based NSs into solid products at the lab scale. This approach was considered appropriate prior to exploring larger-scale equipment, such as high-shear mixers, given the possibility to work with limited NS volume and to perform a preliminary screening of a variety of carriers.

Two hydrophilic binders (i.e., PVP K-30 and PVP/VA 64) were added (2.5 wt% and 5 wt%) to the CN NS to attain the granulation liquid. Although increasing binder concentration would be expected to reduce nanocrystal mobility and collision frequency, the relatively high PVP and PVP/VA 64 amounts used here may also destabilize drug nanocrystal dispersions [52,53,54]. Indeed, competitive adsorption with the primary surfactant stabilizer (i.e., TPGS) might have occurred, leading to altered interfacial composition and less effective steric/surfactant protection. On the other hand, the rise in granulation liquid viscosity can hinder redispersion of loosely associated nanocrystal aggregates formed during processing [55,56]. Therefore, particle size control of the CN NS was carried out immediately after binder addition and repeated after 24 h (Table 3). Interestingly, neither the type nor the concentration of the selected binders affected the dimensional characteristics of CN.

For the carrier screening phase, a broad range of excipients was tested based on two key performance criteria: (i) their intrinsic ability to interact with water (i.e., moisture-uptaking properties), which can affect granule formation, and (ii) their potential impact on the final product phyisio-technological as well as release characteristics, whether granules are used as such or subjected to further processing (e.g., tableting). In this respect, granule-forming agents, which were expected, based on their interaction mechanics with aqueous fluids, to support either immediate or prolonged release of CN, were taken into account. Based on the above-mentioned considerations, the carriers employed in this work are summarized below, together with their key properties as reported in the drug delivery literature:

- EXP: cross-linked sodium starch glycolate, a superdisintegrant known for its pronounced swelling upon contact with water (up to 300 times its volume). This behavior facilitates prompt disintegration and thus immediate drug release. Based on the above-mentioned properties, it is widely used in both direct compression and wet granulation [57,58];
- SP: soy polysaccharides acting as disintegrants. Derived from non-genetically modified soy, they ensure consistent disintegration performance with minimal impact on tablet tensile strength [59,60,61];
- LT: lactose monohydrate, a widely used filler for tablets and capsules. Its cohesive properties make it suitable for both wet and dry granulation processes [62,63];
- ERL and EPO: methacrylate-based polymers traditionally employed in coating processes. More in detail, Eudragit^®^ E is a cationic, pH-dependent polymer soluble in acidic media (pH < 5), which is generally applied on the outer surface of tablets and capsules for taste-masking purposes [64]. More recently, it has also been tested as a processing aid in hot-melt extrusion and 3D printing to produce rapidly dissolving oral dosage forms [65]. On the other hand, Eudragit^®^ RL is a cationic, water-insoluble but highly permeable material, widely used as a pH-independent matrix former and coating polymer to achieve prolonged release systems by modulating water uptake and drug diffusion [66];
- EC: ethylcellulose, water-insoluble cellulose derivative, widely used as a hydrophobic matrix former and coating polymer to control diffusion in prolonged-release oral formulations, and provide moisture-protective coatings for sensitive active drugs [67,68];
- HPMCAS: hydroxypropyl methylcellulose acetate succinate with acetyl and succinyl groups that determine its pH-dependent solubility. Indeed, it is insoluble in gastric fluids, while it swells and dissolves at higher pH values, making it suitable as an enteric coating agent [69,70]. Moreover, it was also demonstrated to be suitable for the preparation of solid dispersions by hot melt extrusion and 3D printing, being able to improve the oral bioavailability of poorly soluble compounds [71,72,73].

### 3.2. Placebo Granules

Initially, placebo granules (formulations detailed in Table 1) were manufactured using a simple water-based binder solution to assess the intrinsic capability of different carriers to absorb water. This assessment was essential to understand how each material handled high amounts of liquid, a relevant aspect given the high-water content of the CN NS. Indeed, by quantifying the amount of water absorbed per wetting cycle before drying (2 h at 40 °C), it was possible to estimate the number of granulation/drying cycles required to reach the desired CN load. Formulations containing SP, HPMCAS, EC, ERL, and EPO required no more than two drying steps, in line with their known water-handling properties. In contrast, the LT-based formulation proved the most challenging, due to the high solubility of lactose. Variations in binder type or concentration generally had a limited impact on the WG process, except for EXP, which showed a shorter drying time when granulated with PVP/VA 64 compared to PVP K-30.

This screening was crucial for defining a viable granulation strategy for each material while avoiding wasting the CN NS. Moreover, it enabled the identification of interesting carrier/binder combinations for the subsequent preparation of CN NS-loaded granules. All the characterization results relevant to placebo formulations are summarized in Table 4.

As highlighted in the table, reference criteria were established to facilitate the identification of the most promising formulations. By way of example, these included the following: (i) “excellent” or “good” flowability, (ii) friability below 15%, and (iii) product yield above 50%. With respect to the above-mentioned key parameters, adequate powder flow was deemed essential for potential downstream processing. Indeed, granules classified as excellent or good according to standard flow indices are generally associated with improved filling capabilities, for instance, of capsule bodies or dies in tableting manufacturing, reduced weight variability, and thus enhanced process robustness [74,75,76]. On the other hand, the friability value selected as a cut-off reflected sufficient mechanical strength of the products to withstand handling, sieving, and subsequent processing without excessive breakage [28,30,77]. Finally, a yield above 50% for the targeted size fraction was considered an acceptable compromise to obtain a sufficiently representative and processable fraction for subsequent characterization and the expected variability of the adopted processing [78,79,80]. Indeed, differences in liquid distribution, kneading intensity, and endpoint determination would produce broader and more variable particle sizes, limiting the proportion of material falling within a narrow sieve cut. In a broader perspective, an increase in yields can be attained when a promising formulation is selected, and the process is transferred to pilot-scale equipment, where optimization of operating parameters (e.g., binder addition, impeller/pan speed, and spray/drying conditions) would be carried out depending on the targeted granule size fraction.

Among the SP-based samples, the use of PVP/VA 64 improved flowability, product yield, and mass loss compared with PVP K-30. LT formulations containing PVP/VA 64 were characterized by higher friability but maintained yields above 50%, while flowability remained comparable to the corresponding batches containing PVP K-30. EC samples showed greater variability: most of them showed yields below 50%, with EC2b as an exception, though associated with higher mass loss. EC1b displayed excellent flowability and acceptable friability, but low overall yield. PVP K-30-based EC products pointed out insufficient yield and, in some cases, pointed out high mass loss or poor flowability. HPMCAS- and EPO-based granules performed consistently, with yields around 80% and mass loss ≤10%. Flowability was slightly improved using PVP/VA 64, particularly in EPO samples. EXP granules showed comparable flowability across batches, while differences were observed in product yield (~50% with PVP/VA 64) and mass loss, which remained below 10% only in 1b and 2b specimens. ERL formulations had analogous, though suboptimal, flowability, high mass loss, and excellent product yield (>90%).

Based on these findings, PVP/VA 64 was selected as the binder for the preparation of drug-loaded granules, testing both the minimum (2.5% *w*/*w*) and the maximum (5% *w*/*w*) binder concentrations.

### 3.3. CN NS-Loaded Granules

Based on the results from the placebo trials, CN NS containing granules were produced (formulations detailed in Table 1) and characterized to evaluate the impact of using the NS as the wetting liquid on the previously defined granules key quality attributes (Table 5).

Overall, the physico-technological characteristics of NS-loaded granules were maintained or even improved, particularly in terms of flowability and mass loss. This behavior can be attributed to the intrinsic physico-chemical characteristics of CN, which might promote stronger inter-particulate bonding and greater granule densification during wet massing and drying, resulting in better flowing granules with higher mechanical resistance [28,81]. Conversely, product yield relevant to the targeted granulometric fraction remained comparable or slightly reduced, as the presence of a crystalline drug does not eliminate the inherent variability of manual WG, as previously discussed [82]. Thus, while the presence of CN can enhance granule quality attributes, it does not necessarily increase the recovery of the specific size fraction undergoing subsequent evaluation [28,83].

**Figure 2:**
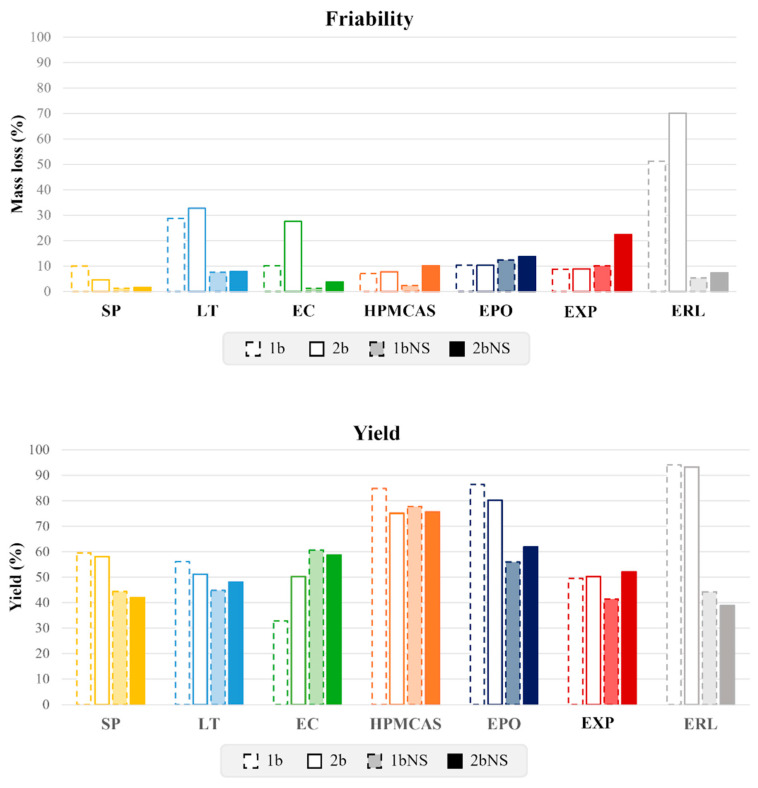
Friability and yield values relevant to placebo and CN NS-loaded granules based on different carriers.

More in detail, WG using the CN NS generally resulted in granules with markedly reduced friability (overall 10%, Figure 2) with respect to their placebo counterparts. EXP2bNS samples were the sole exception, which exhibited a mass loss of approximately 20%. A particularly illustrative example is provided by ERL1b and ERL2b, as placebo formulations showed mass losses of 51% and 70%, respectively, while the corresponding NS-loaded granules displayed values of only 5% and 7%.

Regarding product yield, most NS-loaded granules performed similarly to their placebo equivalents (Figure 1). Indeed, in the majority of cases, yields exceeded 50%, with only a few formulations showing slightly lower values, though never below 40%. The most pronounced deviation was observed for ERL-based samples, which were characterized by a substantial reduction in yield, i.e., approximately 50% lower than the corresponding placebo counterparts.

The use of the CN NS as the granulating liquid did not compromise the flowability of the resulting granules. Except for the LT-based formulations, which exhibited only a slight deviation from their placebo analogues, all drug-containing samples displayed flow properties comparable to the corresponding placebo batches. Notably, EXP1bNS, EXP2bNS, ERL1bNS, and ERL2bNS products even exhibited improved flowability.

#### 3.3.1. Drug Content and Nanocrystals Redispersibility

Evaluating the actual CN content in granules was considered a critical step in the screening process. Indeed, it would allow us to verify whether the drug was effectively retained during the WG process and whether any major loss occurred. Moreover, the RDI of the CN nanocrystals from granules was evaluated in comparison with that of the reference CN NS, to ensure that the manufacturing processes, and especially the drying phase, did not alter the original particle size of drug nanocrystals (Table 6). RDI values approaching 1.0 indicated optimal redispersion of nanocrystals from the granules, with values < 1.20 being considered acceptable [12,84].

Analysis of CN content confirmed an overall high loading efficiency, with most formulations showing values above 95%. All granules exhibited acceptable RDIs, indicating an effective redispersion of the nanocrystals and demonstrating that the solidification process did not alter the original CN NS particle size. Minor deviations were observed for HPMCAS-based samples. However, values falling outside the acceptable range are likely attributable to the presence of insoluble carrier-derived particles, which are larger in size but indistinguishable from the CN nanocrystals using the analytical technique employed. Indeed, sizing techniques commonly used to determine RDI measure the overall scattering/diffraction signal of the sample, being unable to discriminate primary drug nanocrystals from larger, insoluble carrier-derived fragments or aggregates. In this respect, the presence of even a small fraction of larger carrier particles will shift the measured distribution and can produce apparent out-of-range RDI values [85,86]. Thus, incomplete breakup of agglomerates during the redispersion step or the co-presence of carrier-derived insoluble particulates can increase the apparent fraction of large particles despite intact CN nanocrystals being present [87,88]. Method-related aspects might also amplify this effect: Indeed, DLS is intensity-weighted and, therefore, disproportionately emphasizes larger scatterers, so minor amounts of debris or aggregates can dominate the reported distributions [85,89,90]. For these reasons, RDI values slightly outside the predefined acceptance range could result from the co-existence of insoluble carrier fragments and known limitations of the sizing methods, rather than indicating irreversible growth of CN nanoscrystals.

#### 3.3.2. In Vitro Performance

**Figure 3:**
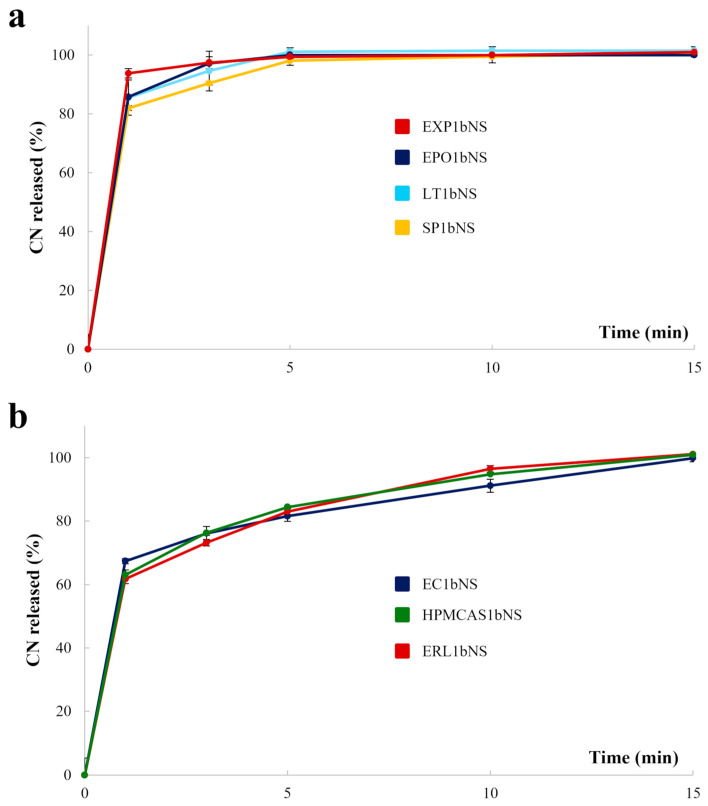
Release profiles relevant to CN NS-loaded granules based on different known from the literature for providing either (**a**) immediate or (**b**) modified-release performances.

Assessing the release behavior of CN NS-loaded granules was a pivotal part of the characterization phase. Indeed, in products containing drug nanocrystals, dissolution performance serves not only as an indicator of the WG efficiency but also provides valuable insights into two key aspects: (i) whether the selected solidification process preserves the nanoscale particle size of the drug, allowing the granules to exhibit a release pattern comparable to the original NS, and (ii) the capability of the selected carriers to meet their expected release-modulation function (e.g., ensuring controlled release even when dealing with granules containing drug nanocrystals that, due to their size, can easily escape entrapment within the polymeric matrix). Representative release profiles relevant to granules containing the lowest binder concentration (i.e., 1bNS formulations) are shown in Figure 3. Indeed, formulations with the highest binder concentration (i.e., 2bNs) pointed out analogous release patterns.

Granules based on EXP and EPO released nearly 100% of the conveyed CN within 3 min, while those containing LT and SP reached a plateau within 5 min. This immediate release behavior was expected, given the prompt solubility or disintegration ability of these carriers and considering that the CN NS characteristics were not altered by the WG process. Unexpectedly, granules prepared starting from EC, HPMCAS, and ERL, which are polymers traditionally used in the development of controlled-release systems, also release the entire drug content within 15 min. Given these results, the WG process likely did not achieve sufficient granule densification nor an effective embedding of the CN nanocrystals within a continuous polymer matrix [28]. Therefore, the carriers were unable to give rise to a coherent diffusion-controlling barrier, and the drug remained mainly deposited on the granule surface, resulting in rapid dissolution. A similar behavior in polymer-based controlled-release systems, arising from inadequate matrix formation or limited polymer–drug integration, has already been described [68,91,92,93,94]. In addition, the rapid release was probably further amplified by the intrinsic properties of CN nanocrystals [87,88]. Their very high specific surface area markedly accelerated dissolution, which can dominate over the release-modifying capability imparted by the carriers, particulate for dosage forms with insufficient embedding and limited density, thus high porosity. In the present case, this phenomenon could result in a particularly pronounced effect under acidic sink conditions, which further accelerate CN dissolution. Overall, the combination of (i) insufficient polymer structuring resulting from the manual WG process and (ii) the intrinsically fast dissolution of CN nanocrystals in HCl 0.1 N likely masked the release-controlling properties of the selected polymers, ultimately resulting in immediate-release profiles.

### 3.4. Tableting

As a further assessment of their performance, granules were used to prepare mini-tablets, which, due to their size, can still be considered as multi-particulate systems. Besides exploring their suitability for downstream processing, this step helped in evaluating whether (i) the application of mechanical stress during tableting would compromise the physico-chemical and dimensional properties of the CN nanocrystals conveyed, and (ii) the densification achieved during compression could enhance the release behavior of products intended for controlled release. To this end, EC-based granules were selected as a model system. Mini-tablets were produced starting from granules having the highest binder concentration (i.e., EC2bNS), which were blended with either Mg St alone or in combination with HPMC K4M (Table 2), which was used as a matrix-forming polymer. All tablets exhibited acceptable weight uniformity and comparable average thickness (Table 7). On the other hand, prototypes containing HPMC K4M showed reduced mechanical resistance.

#### 3.4.1. In Vitro Performance

**Figure 4:**
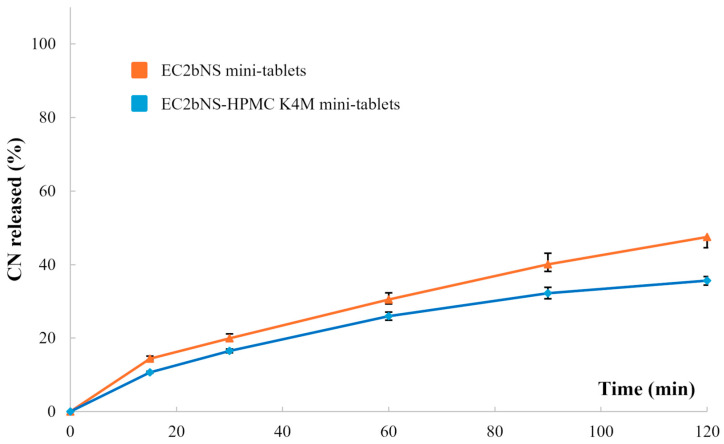
Release profiles relevant to EC2b mini-tablets formulated with and without HPMC K4M.

Assessing the release performance of EC-based mini-tablets allowed us to estimate whether the densification entailed by the tableting process and the presence of high viscosity HPMC effectively slowed down the release rate of the starting granules (Figure 4).

The resulting dissolution profiles clearly demonstrated that granule compaction effectively modified the release pattern compared with the corresponding uncompressed products (Figure 3). Specifically, EC2bNS prototypes exhibited a slower release rate, releasing approximately 50% of the CN conveyed within 2 h. Adding HPMC K4M further reduced the release rate, with mini-tablets releasing approximately 35% of the loaded CN over the same timeframe. These data highlighted the positive influence of a hydrophilic matrix-forming agent to strengthen the structural integrity of the mini-tablet and to enhance diffusional control, in line with the well-established role of HPMC in modulating drug release from matrices [91,95,96]. Indeed, hydrophilic swellable/erodible polymers are well known for their ability to give rise to viscous gel layers upon contact with aqueous media, which slow down drug diffusion and counteract the rapid dissolution typically associated with nanocrystal-based formulations [97].

These findings also support the hypothesis that the initial granules did not achieve sufficient densification during manual WG. On the other hand, the additional mechanical compression involved in tablet manufacturing reduced porosity and promoted intimate contact between the polymer and the nanocrystals, enabling the formation of a more coherent matrix able to better control the release rate [94].

Overall, the in vitro performance results confirmed that granules, when used as intermediates, can provide the desired prolonged-release profile thanks to the densification impaired by tableting. Moreover, the control on the release rate can be further fine-tuned by incorporating additional release-controlling polymers.

#### 3.4.2. Nanocrystals’ Redispersibility

Assessing the preservation of the CN nanoscale dimensions was essential to determine the actual impact of an additional technological steps (e.g., tableting) on the original drug particle size (Table 8).

In this case as well, the measured dimensional values were found to be consistent with those relevant to the starting CN NS, with RDI data falling within the optimal range. More in detail, the CN nanocrystals were effectively redispersed by the EC2bNS mini-tablets. On the other hand, EC2bNS-HPMC K4M samples displayed slightly higher-than-expected measurements.

## 4. Conclusions

This work served as a proof-of-concept to assess, at the lab scale, the feasibility of converting a CN-containing NS, which was representative of a poorly water-soluble BCS class II drug, into solid dosage forms for oral administration while preserving the nano-scale dimensions and the solid-state characteristics of the drug. The WG process proved effective in producing granules with suitable physio-technological properties. With respect to the in vitro performance, they showed a prompt CN release, with no capability of controlling the release rate. Considering the use of carriers traditionally used in the development of controlled-release systems, this finding was unexpected. Achieving prolonged-release behavior will likely require WG technologies with greater densification efficiency and improved incorporation of drug nanocrystals. Moreover, granules were successfully processed into mini-tablets, which, as expected, enabled modulation of CN release by decreasing product porosity and reducing the surface area exposed to aqueous media, ultimately allowing slower release patterns to be achieved. Importantly, neither granulation nor tableting altered the CN NS size characteristics, which are essential for the biopharmaceutical performance of the final product. Overall, the data collected in this work confirmed that using an NS as the binding liquid in WG is an effective strategy for obtaining multi-particulate systems with excellent technological properties, suitable for direct subdivision (e.g., into capsules or sachets) or for downstream processing. This approach results in a versatile product for different uses and, in principle, is suitable for supporting a portfolio of release kinetics. As such, it could be particularly valuable during the early screening of new BCS class II drug candidates for future medicinal products, accelerating development while reducing both relevant time and costs.

## Figures and Tables

**Table 1:** (**a**) Overview of the formulation codes used in the study, including binder type and concentration, carrier material, and presence or absence of CN NS, and (**b**) formulation composition of placebo and CN NS-loaded granules.

a									
Abbreviation	Description								
1a	PVP K-30 binder at 2.5% (placebo)								
2a	PVP K-30 binder at 5% (placebo)								
1b	PVP/VA 64 binder at 2.5% (placebo)								
2b	PVP/VA 64 binder at 5% (placebo)								
1aNS, 2aNS, 1bNS, 2bNS	Granulation liquids containing CN NS								
SP1a, SP2a, SP1b, SP2b	Soy polysaccharide granules with binder variants (placebo)								
LT1a, LT2a, LT1b, LT2b	Lactose monohydrate granules with binder variants (placebo)								
EC1a, EC2a, EC1b, EC2b	Ethyl cellulose granules with binder variants (placebo)								
EPO1a, EPO2a, EPO1b, EPO2b	Eudragit^®^ E PO granules with binder variants (placebo)								
ERL1a, ERL2a, ERL1b, ERL2b	Eudragit^®^ RL PO granules with binder variants (placebo)								
EXP1a, EXP2a, EXP1b, EXP2b	Sodium starch glycolate granules with binder variants (placebo)								
HPMCAS1a, HPMCAS2a, HPMCAS1b, HPMCAS2b	Hydroxypropyl methylcellulose acetate succinate granules with binder variants (placebo)								
SP1bNS, SP2bNS	Soy polysaccharide granules with PVP/VA 64 binder variants containing CN NS								
LT1bNS, LT2bNS	Lactose monohydrate granules with PVP/VA 64 binder variants containing CN NS								
EC1bNS, EC2bNS	Ethyl cellulose granules with PVP/VA 64 binder variants containing CN NS								
EPO1bNS, EPO2bNS	Eudragit^®^ E PO granules with PVP/VA 64 binder variants containing CN NS								
ERL1bNS, ERL2bNS	Eudragit^®^ RL PO granules with PVP/VA 64 binder variants containing CN NS								
EXP1bNS, EXP2bNS	Sodium starch glycolate granules with PVP/VA 64 binder variants containing CN NS								
HPMCAS1bNS, HPMCAS2bNS	Hydroxypropyl methylcellulose acetate succinate granules with PVP/VA 64 binder variants containing CN NS								
**b**									
Formulation code		1a	2a	1b	2b	1aNS	2aNS	1bNS	2bNS
Carrier *, %		85	82.5	85	82.5	85	82.5	85	82.5
CN, %		-				10			
TPGS, %		-				2.5			
Binder, %	PVP K-30	2.5	5	-	-	2.5	5	-	-
	PVP/VA 64	-	-	2.5	5	-	-	2.5	5

* alternatives: SP, LT, EC, EPO, ERL, EXP, HPMCAS.

**Table 2:** Mini-tablet compositions (*w*/*w* %).

	EC2bNS Mini-Tablet	EC2bNS-HPMC K4M Mini-Tablet
Granulate (%)	98.5	49.25
Mg St (%)	1.5	1.5
HPMC K4M (%)	0	49.25

**Table 3:** Z-avg and PdI relevant to the CN NS supplemented with different binders at increasing concentrations, measured immediately after preparation (t_0_) and after 24 h (t_24h_).

	Dimensional Analysis	t	t_24_
1aNS	Z-avg, nm ± SD	346.37 ± 0.50	347.73 ± 5.16
	PdI	0.181 ± 0.026	0.259 ± 0.015
1bNS	Z-avg, nm ± SD	353.40 ± 1.01	350.27 ± 2.90
	PdI	0.176 ± 0.008	0.297 ± 0.013
2aNS	Z-avg, nm ± SD	338.40 ± 2.71	341.07 ± 0.76
	PdI	0.174 ± 0.004	0.261 ± 0.015
2bNS	Z-avg, nm ± SD	342.47 ± 2.05	345.53 ± 4.22
	PdI	0.218 ± 0.021	0.209 ± 0.012

**Table 4:** Physico-technological characteristics of placebo granules (355–500 µm size fraction).

	Yield, %	Friability, % of Mass Loss	$\rho_{b}$, g/mL	CI, %	Flowability *
SP1a	69.11	30.10	0.319	20.63	Passable
SP2a	48.86	30.05	0.271	13.51	Good
SP1b	59.58	10.04	0.379	13.21	Good
SP2b	58.11	4.63	0.386	13.46	Good
LT1a	40.23	5.47	0.469	18.60	Fair
LT2a	43.77	9.52	0.489	14.63	Good
LT1b	56.04	28.77	0.590	11.76	Good
LT2b	51.15	32.82	0.445	15.56	Good
EC1a	27.66	23.46	0.410	11.90	Good
EC2a	42.00	10.27	0.400	18.00	Fair
EC1b	32.82	10.12	0.427	8.51	Excellent
EC2b	50.19	27.55	0.392	13.73	Good
HPMCAS1a	88.63	2.50	0.477	16.67	Fair
HPMCAS2a	84.07	1.02	0.500	15.00	Good
HPMCAS1b	84.87	7.06	0.501	12.50	Good
HPMCAS2b	75.07	7.82	0.488	12.20	Good
EPO1a	88.43	8.98	0.445	17.78	Fair
EPO2a	81.41	8.80	0.400	18.00	Fair
EPO1b	86.43	10.29	0.501	10.00	Excellent
EPO2b	80.22	10.31	0.456	13.64	Good
EXP1a	29.13	12.52	0.485	13.89	Good
EXP2a	51.67	36.41	0.455	15.91	Fair
EXP1b	49.55	8.77	0.417	16.67	Fair
EXP2b	50.20	8.93	0.440	15.22	Good
ERL1a	91.16	36.97	0.36	21.43	Passable
ERL2a	82.61	44.42	0.27	24.32	Passable
ERL1b	94.16	51.27	0.33	25.00	Passable
ERL2b	93.21	70.07	0.34	23.73	Passable

* defined based on Eur. Ph. XI Ed., 2.9.36—Powder flow.

**Table 5:** Physio-technological characteristics of CN NS-loaded granules (355–500 µm particle size fraction).

	Yield, %	D_50_, µm ± SD	Friability, % of Mass Loss	$\rho_{b}$	CI, %	Flowability *
SP1bNS	44.32	480 ± 3.2	1.40	0.334	15.38	Good
SP2bNS	41.97	478 ± 1.2	1.64	0.352	14.00	Good
LT1bNS	44.92	495 ± 9.0	7.55	0.557	16.67	Fair
LT2bNS	48.17	505 ± 2.3	7.94	0.557	16.67	Fair
EC1bNS	60.68	482 ± 1.7	1.40	0.392	9.80	Excellent
EC2bNS	58.71	466 ± 2.6	3.85	0.394	11.76	Good
HPMCAS1bNS	77.85	504 ± 6.2	2.42	0.513	10.26	Excellent
HPMCAS2bNS	75.71	517 ± 9.6	10.16	0.477	11.90	Good
EPO1bNS	55.98	529 ± 14.2	12.52	0.476	14.29	Good
EPO2bNS	61.93	519 ± 6.1	13.84	0.500	12.50	Good
EXP1bNS	41.41	425 ± 11.7	10.11	0.400	14.00	Good
EXP2bNS	52.06	490 ± 3.2	22.52	0.510	10.26	Excellent
ERL1bNS	44.28	430 ± 12.5	5.33	0.390	17.65	Fair
ERL2bNS	38.87	455 ± 5.5	7.34	0.390	13.46	Good

* defined based on Eur. Ph. XI Ed., 2.9.36—Powder flow.

**Table 6:** Drug content, Z-avg, PdI, and RDI values relevant to CN NS-loaded granules based on different carriers.

	CN Content, % (CV)	Z-avg, nm ± SD	PdI ± SD	RDI
SP1bNS	105.05 (1.97)	346.4 ± 4.42	0.210 ± 0.01	1.007
SP2bNS	99.02 (11.36)	345.1 ± 7.29	0.212 ± 0.02	1.003
EC1bNS	93.56 (1.48)	355.8 ± 3.59	0.239 ± 0.01	1.034
EC2bNS	95.06 (4.72)	320.0 ± 0.80	0.213 ± 0.01	0.930
HPMCAS1bNS	97.68 (2.91)	587.1 ± 6.84	0.382 ± 0.03	1.707
HPMCAS2bNS	99.04 (4.07)	475.9 ± 5.35	0.302 ± 0.04	1.383
LT1bNS	98.94 (15.87)	388.0 ± 4.76	0.345 ± 0.04	1.128
LT2bNS	100.61 (2.52)	344.5 ± 0.85	0.204 ± 0.02	1.001
EXP1bNS	108.89 (5.76)	357.2 ± 4.30	0.185 ± 0.02	1.038
EXP2bNS	104.13 (3.48)	349.3 ± 6.92	0.163 ± 0.01	1.015
EPO1bNS	94.51 (7.75)	401.4 ± 6.78	0.298 ± 0.03	1.167
EPO2bNS	103.7 (7.87)	392.5 ± 5.00	0.278 ± 0.02	1.141
ERL1bNS	101.72 (2.16)	379.3 ± 5.22	0.241 ± 0.01	1.103
ERL2bNS	103.96 (3.96)	415.6 ± 1.32	0.246 ± 0.02	1.208

**Table 7:** Tableting parameters and physico-technological characteristics of mini-tablets based on different formulations.

	Weight, mg ± SD	Compression Force, kN ± SD		Ejection Force, kN ± SD	Thickness, mm ± SD	Hardness, N ± SD
		Upper Punch	Lower Punch			
EC2bNS mini-tablet	25.20 ± 2.20	4.87 ± 0.16	4.91 ± 0.15	21.06 ± 1.15	2.30 ± 0.06	14.3 ± 1.53
EC2bNS-HPMC K4M mini-tablet	25.32 ± 2.12	3.78 ± 0.03	3.81 ± 0.03	23.36 ± 0.81	2.16 ± 0.02	4.0 ± 0.52

**Table 8:** Z-avg, PdI, and RDI values relevant to CN NS-loaded mini-tablets based on different formulations.

Sample	Z-avg, nm ± SD	PdI ± SD	RDI
EC2bNS mini-tablets	368.8 ± 8.44	0.382 ± 0.06	1.072
EC2bNS-HPMC K4M mini-tablets	443.0 ± 10.08	0.395 ± 0.03	1.288

## Data Availability

The original contributions presented in this study are included in the article. Further inquiries can be directed to the corresponding author.

## Abbreviations

BCS	Biopharmaceutical classification system
CI	Compressibility index
CN	Cinnarizine
CN NS	Cinnarizine-containing nanosuspension
D_50_	Median particle diameter
DLS	Dynamic light scattering
EC	Ethyl cellulose, i.e., Ethocel™ Standard 10 Premium
EPO	Polymethacrylate copolymer, i.e., Eudragit^®^ E PO
ERL	Polymethacrylate copolymer, i.e., Eudragit^®^ RL PO
EXP	Sodium starch glycolate, i.e., Explotab^®^ CLV
HPLC	High-performance liquid chromatography
HPMCAS	Hydroxypropyl methylcellulose acetate succinate, i.e., AQOAT^®^ AS
LT	Lactose monohydrate, i.e., GranuLac^®^ 200
NCE	New chemical entity
NS	Nanosuspension
PdI	Polydispersity index
PSD	Particle size distribution
RDI	Redispersibility index
SP	Soy polysaccharides, i.e., Emcosoy^®^
USP	United States pharmacopoeia
UV–Vis	Ultraviolet–visible spectroscopy
WG	Wet granulation
Z-avg	Z-average hydrodynamic diameter

## References

[1] Di Stefano A. (2023). Nanotechnology in targeted drug delivery. Int. J. Mol. Sci..

[2] Zhuo Y., Zhao G., Zhang Y. (2024). enhancing drug solubility, bioavailability, and targeted therapeutic applications through magnetic nanoparticles. Molecules.

[3] Jacob S., Kather F.S., Boddu S.H.S., Attimarad M., Nair A.B. (2025). Nanosuspension innovations: Expanding horizons in drug delivery techniques. Pharmaceutics.

[4] Malamatari M., Somavarapu S., Taylor K.M.G., Buckton G. (2016). Solidification of nanosuspensions for the production of solid oral dosage forms and inhalable dry powders. Expert Opin. Drug Deliv..

[5] Patel V., Agrawal Y. (2011). Nanosuspension: An approach to enhance solubility of drugs. J. Adv. Pharm. Technol. Res..

[6] Chavda V.P., Vaghela D.A., Solanki H.K., Balar P.C., Modi S., Gogoi N.R. (2025). Nanosuspensions: A new era of targeted therapeutics. J. Drug Deliv. Sci. Technol..

[7] Jacob S., Nair A.B., Shah J. (2020). Emerging role of nanosuspensions in drug delivery systems. Biomater. Res..

[8] Hanwate R.M., Babu R.H., Wadhave A.A., Mishra V. (2025). Advancements in nanosuspension technology for drug delivery. Biomed. Mat. Devices.

[9] Leone F., Cavalli R. (2015). Drug nanosuspensions: A ZIP tool between traditional and innovative pharmaceutical formulations. Expert Opin. Drug Deliv..

[10] Aghrbi I., Fülöp V., Jakab G., Kállai-Szabó N., Balogh E., Antal I. (2021). Nanosuspension with improved saturated solubility and dissolution rate of cilostazol and effect of solidification on stability. J. Drug Deliv. Sci. Technol..

[11] Patel N.C., Patel H.A. (2022). A recent solidification approach for nanosuspension: Formulation, optimisation and evaluation of canagliflozin immediate release pellets. Folia Medica.

[12] Zhang X., Guan J., Ni R., Li L.C., Mao S. (2014). Preparation and solidification of redispersible nanosuspensions. J. Pharm. Sci..

[13] Tsiaxerli A., Vardaka E., Moutroupidis C., Taylor K.M.G., Kachrimanis K., Malamatari M. (2024). Updates on the conversion of nanosuspensions to solid oral dosage forms. J. Food Drug Anal..

[14] Malamatari M., Charisi A., Malamataris S., Kachrimanis K., Nikolakakis I. (2020). Spray Drying for the Preparation of Nanoparticle-Based Drug Formulations as Dry Powders for Inhalation. Processes.

[15] Vauthier C., Bouchemal K. (2008). Methods for the Preparation and Manufacture of Polymeric Nanoparticles. Pharm. Res..

[16] Jakubowska E., Bielejewski M., Milanowski B., Lulek J. (2022). Freeze-drying of drug nanosuspension—Study of formulation and processing factors for the optimization and characterization of redispersible cilostazol nanocrystals. J. Drug Deliv. Sci. Technol..

[17] Pardeshi R., Deshmukh N.S., Telange D.R., Nangare S.N., Sonar Y.Y., Lakade S.H., Harde M.T., Pardeshi C.V., Gholap A., Deshmukh P.K. (2023). Process development and quality attributes for the freeze-drying process in pharmaceuticals, biopharmaceuticals and nanomedicine delivery: A state-of-the-art review. Future J. Pharm. Sci..

[18] Ragucci E., Uboldi M., Sobczuk A., Facchetti G., Melocchi A., Serratoni M., Zema L. (2025). Feasibility of hot melt extrusion in converting water-based nanosuspensions into solid dosage forms. Pharmaceutics.

[19] Baumgartner R., Eitzlmayr A., Matsko N., Tetyczka C., Khinast J., Roblegg E. (2014). Nano-extrusion: A promising tool for continuous manufacturing of solid nano-formulations. Int. J. Pharm..

[20] Pınar S.G., Oktay A.N., Karaküçük A.E., Çelebi N. (2023). Formulation strategies of nanosuspensions for various administration routes. Pharmaceutics.

[21] Tran B.N., Tran H.T., Le G.T., Tran H.P., Le K.N., Do H.H., Dao A.H., Nguyen C.N. (2023). Solidifying fenofibrate nanocrystal suspension: A scalable approach via granulation method. J. Nanomater..

[22] Faure A., York P., Rowe R.C. (2001). Process control and scale-up of pharmaceutical wet granulation processes: A review. Eur. J. Pharm. Biopharm..

[23] Giry K., Genty M., Viana M., Wuthrich P., Chulia D. (2006). Multiphase versus single pot granulation process: Influence of process and granulation parameters on granules properties. Drug Dev. Ind. Pharm..

[24] Szulc A., Skotnicka E., Gupta M.K., Królczyk J.B. (2024). Powder agglomeration processes of bulk materials—A state of the art review on different granulation methods and applications. Powder Technol..

[25] Wang B., Sun X., Xiang J., Guo X., Cheng Z., Liu W., Tan S. (2022). A critical review on granulation of pharmaceuticals and excipients: Principle, analysis and typical applications. Powder Technol..

[26] Vadaga A.K., Gudla S.S., Nareboina G.S.K., Gubbala H., Golla B. (2024). Comprehensive review on modern techniques of granulation in pharmaceutical solid dosage forms. Intell. Pharm..

[27] Suresh P., Sreedhar I., Vaidhiswaran R., Venugopal A. (2017). A comprehensive review on process and engineering aspects of pharmaceutical wet granulation. Chem. Eng. J..

[28] Iveson S.M., Litster J.D., Hapgood K., Ennis B.J. (2001). Nucleation, growth and breakage phenomena in agitated wet granulation processes: A review. Powder Technol..

[29] Shanmugam S. (2015). Granulation techniques and technologies: Recent progresses. Bioimpacts.

[30] Parikh M. (2021). Handbook of Pharmaceutical Granulation Technology.

[31] Kittikunakorn N., Liu T., Zhang F. (2020). Twin-screw melt granulation: Current progress and challenges. Int. J. Pharm..

[32] Liu B., Wang J., Zeng J., Zhao L., Wang Y., Feng Y., Du R. (2021). A review of high shear wet granulation for better process understanding, control and product development. Powder Technol..

[33] Liu T., Kittikunakorn N., Zhang Y., Zhang F. (2021). Mechanisms of twin screw melt granulation. J. Drug Deliv. Sci. Technol..

[34] Thompson M.R. (2015). Twin screw granulation- Review of current progress. Drug Dev. Ind. Pharm..

[35] Zhang Y., Liu T., Kashani S., Zhang F. (2021). A review of twin screw wet granulation mechanisms in relation to granule attributes. Drug Dev. Ind. Pharm..

[36] Loh Z.H., Er D.Z.L., Chan L.W., Liew C.V., Heng P.W.S. (2011). Spray granulation for drug formulation. Expert Opin. Drug Deliv..

[37] Rubino O.P. (1999). Fluid-bed technology: Overview and criteria for process selection. Pharm. Technol..

[38] Burggraeve A., Monteyne T., Vervaet C., Remon J.P., Beer T.D. (2013). Process analytical tools for monitoring, understanding, and control of pharmaceutical fluidized bed granulation: A review. Eur. J. Pharm. Biopharm..

[39] Miwa A., Yajima T., Itai S. (2000). Prediction of suitable amount of water addition for wet granulation. Int. J. Pharm..

[40] Litster J., Ennis B. (2004). The Science and Engineering of Granulation Processes.

[41] Amidon G.L., Lennernäs H., Shah V.P., Crison J.R. (1995). A theoretical basis for a biopharmaceutic drug classification: The correlation of in vitro drug product dissolution and in vivo bioavailability. Pharm. Res..

[42] Maghsoodi M., Nokhodchi A., Oskuei M.A., Heidari S. (2019). Formulation of cinnarizine for stabilization of its physiologically generated supersaturation. AAPS PharmSciTech.

[43] Raghuvanshi S., Pathak K. (2014). Recent advances in delivery systems and therapeutics of cinnarizine: A poorly water soluble drug with absorption window in stomach. J. Drug Deliv..

[44] Kirtane M.V., Bhandari A., Narang P., Santani R. (2019). Cinnarizine: A contemporary review. Indian J. Otolaryngol. Head Neck Surg..

[45] Shi S., Chen H., Lin X., Tang X. (2010). Pharmacokinetics, tissue distribution and safety of cinnarizine delivered in lipid emulsion. Int. J. Pharm..

[46] Köster C., Pohl S., Kleinebudde P. (2021). Evaluation of Binders in Twin-Screw Wet Granulation. Pharmaceutics.

[47] Kayaert P., Van den Mooter G. (2012). Is the amorphous fraction of a dried nanosuspension caused by milling or by drying? A case study with Naproxen and Cinnarizine. Eur. J. Pharm. Biopharm..

[48] Arndt O.-R., Baggio R., Adam A.K., Harting J., Franceschinis E., Kleinebudde P. (2018). Impact of Different Dry and Wet Granulation Techniques on Granule and Tablet Properties: A Comparative Study. J. Pharm. Sci..

[49] Patel B., Patel J., Thakor R., Rajput G., Patel K. (2010). Improvement of solubility of cinnarizine by using solid dispersion technique. Int. Res. J. Pharm..

[50] Shakeel F., Kazi M., Alanazi F.K., Alam P. (2021). Solubility of cinnarizine in (Transcutol + water) mixtures: Determination, Hansen solubility parameters, correlation, and thermodynamics. Molecules.

[51] Wewers M., Czyz S., Finke J.H., John E., Van Eerdenbrugh B., Juhnke M., Bunjes H., Kwade A. (2020). Influence of formulation parameters on redispersibility of naproxen nanoparticles from granules produced in a fluidized bed process. Pharmaceutics.

[52] Attia L., Nguyen D., Gokhale D., Zheng T., Doyle P.S. (2024). Surfactant–Polymer Complexation and Competition on Drug Nanocrystal Surfaces Control Crystallinity. ACS Appl. Mater. Interfaces.

[53] Hill C., Abdullahi W., Crossman M., Griffiths P.C. (2022). Using Polymer–Surfactant Charge Ratio to Control Synergistic Flocculation of Anionic Particulate Dispersions. Polymers.

[54] Smits J., Giri R.P., Shen C., Mendonc D., Murphy B., Huber P., Rezwan K., Maas M. (2021). Synergistic and Competitive Adsorption of Hydrophilic Nanoparticles and Oil-Soluble Surfactants at the Oil−Water Interface. Langmuir.

[55] Guan W., Ma Y., Ding S., Liu Y., Song Z., Liu X., Tang L., Wang Y. (2022). The technology for improving stability of nanosuspensions in drug delivery. J. Nanopart. Res..

[56] Patravale V.B., Date A.A., Kulkarni R.M. (2004). Nanosuspensions: A promising drug delivery strategy. J. Pharm. Pharmacol..

[57] Kelemen A., Szöllősi A., Zsoter A., Pintye K., Török C., Erős I. (2001). Measurement of the swelling force of some sodium starch glycolate products with new software. Hung. J. Ind. Chem..

[58] Melocchi A., Loreti G., Del Curto M.D., Maroni A., Gazzaniga A., Zema L. (2015). Evaluation of hot-melt extrusion and injection molding for continuous manufacturing of immediate-release tablets. J. Pharm. Sci..

[59] Hosny K.M., Mosli H.A., Hassan A. (2015). Soy polysaccharide as a novel superdisintegrant in sildenafil citrate sublingual tablets: Preparation, characterization, and in vivo evaluation. Drug Des. Dev. Ther..

[60] Joseph K., Premaletha K. (2021). Natural Superdisintegrants for the Formulation of Orally Disintegrating Tablets. Int. J. Res. Rev..

[61] Ursekar B.M., Soni P.S., Date A.A., Nagarsenker M.S. (2012). Characterization of soy polysaccharide and its in vitro and in vivo evaluation for application in colon drug delivery. AAPS PharmSciTech.

[62] Patel P., Telange D., Sharma N. (2011). Comparison of different granulation techniques for lactose monohydrate. Int. J. Pharm. Sci. Drug Res..

[63] Shi C., Zhao H., Fang Y., Shen L., Zhao L. (2023). Lactose in tablets: Functionality, critical material attributes, applications, modifications and co-processed excipients. Drug Discov. Today.

[64] Thakral S., Thakral N.K., Majumdar D.K. (2013). Eudragit^®^: A technology evaluation. Expert Opin. Drug Deliv..

[65] Santos J.D., da Silva G.S., Velho M.C., Beck R.C.R. (2021). Eudragit^®^: A versatile family of polymers for hot melt extrusion and 3d printing processes in pharmaceutics. Pharmaceutics.

[66] Nikam A., Sahoo P.R., Musale S., Pagar R.R., Paiva A.C., Giram P.S. (2023). A systematic overview of Eudragit^®^ based copolymer for smart healthcare. Pharmaceutics.

[67] Mehta R.Y., Missaghi S., Tiwari S.B., Rajabi A.R. (2014). Application of ethylcellulose coating to hydrophilic matrices: A strategy to modulate drug release profile and reduce drug release variability. AAPS PharmSciTech.

[68] Cifuentes C., Aguilar A., Rajabi A.R., Caraballo I. (2013). Critical points in ethylcellulose matrices: Influence of the polymer, drug and filler properties. Acta Pharm..

[69] Choudhari M., Damle S., Saha R.N., Dubey S.K., Singhvi G. (2023). Emerging Applications of Hydroxypropyl Methylcellulose Acetate Succinate: Different Aspects in Drug Delivery and Its Commercial Potential. AAPS PharmSciTech.

[70] Tanno F.K., Sakuma S., Masaoka Y., Kataoka M., Kozaki T., Kamaguchi R., Ikeda Y., Kokubo H., Yamashita S. (2008). Site-specific drug delivery to the middle region of the small intestine by application of enteric coating with hypromellose acetate succinate (HPMCAS). J. Pharm. Sci..

[71] Corrie L., Ajjarapu S., Banda S., Parvathaneni M., Bolla P.K., Kommineni N. (2023). HPMCAS-based amorphous solid dispersions in clinic: A review on manufacturing techniques (hot melt extrusion and spray drying), Marketed Products and Patents. Materials.

[72] Obara S., Tanno F.K., Sarode A. (2013). Properties and applications of hypromellose acetate succinate (HPMCAS) for solubility enhancement using melt extrusion. AAPS Adv. Pharm. Sci. Ser..

[73] Tanno F., Nishiyama Y., Kokubo H., Obara S. (2004). Evaluation of hypromellose acetate succinate (HPMCAS) as a carrier in solid dispersions. Drug Dev. Ind. Pharm..

[74] Shan R.B., Tawakkul M.A., Khan M.A. (2008). Comparative evaluation of flow for pharmaceutical powders and granules. AAPS PharmSciTech.

[75] Pradhan S., Dubey N., Shukla S.S., Pandey R.K., Gidwani B. (2023). A review of the fundamentals of pharmaceutical granulation technology. Int. J. Pharm. Phytopharm. Res..

[76] Shah D.S., Moravkar K.K., Jha D.K., Lonkar V., Amin P.D., Chalikwar S.S. (2023). A concise summary of powder processing methodologies for flow enhancement. Heliyon.

[77] Casian T., Iurian S., Gâvan A., Porfire A., Pop A.L., Crișan S., Pușcaș A.M., Tomuță I. (2022). In-Depth Understanding of Granule Compression Behavior under Variable Raw Material and Processing Conditions. Pharmaceutics.

[78] Kumar A., Radl S., Gernaey K.V., De Beer T., Nopens I. (2021). Particle-Scale Modeling to Understand Liquid Distribution in Twin-Screw Wet Granulation. Pharmaceutics.

[79] Mackaplow M.B., Rosen L.A., Michaels J.N. (2000). Effect of primary particle size on granule growth and endpoint determination in high-shear wet granulation. Powder Technol..

[80] Thapa P., Choi D.H., Kim M.S., Jeong S.H. (2018). Effects of granulation process variables on the physical properties of dosage forms by combination of experimental design and principal component analysis. Asian J. Pharm. Sci..

[81] Salman A.D., Hounslow M.J., Seville J.P.K. (2007). Handbook of Powder Technology, Volume 11: Granulation.

[82] Ennis B.J., Tardos G., Pfeffer R. (1991). A microlevel-based characterization of granulation phenomena. Powder Technol..

[83] Iveson S.M., Litster J.D., Ennis B.J. (1996). Fundamental studies of granule consolidation Part 1: Effects of binder content and binder viscosity. Powder Technol..

[84] Wewers M., Finke J.H., Czyz S., Van Eerdenbrugh B., John E., Büch G., Juhnke M., Bunjes H., Kwade A. (2022). Evaluation of the formulation parameter-dependent redispersibility of api nanoparticles from fluid bed granules. Pharmaceutics.

[85] Farkas N., Kramar J.A. (2021). Dynamic Light Scattering Distributions by Any Means. J. Nanopart. Res..

[86] Jia Z., Li J., Gao L., Yang D., Kanaev A. (2023). Dynamic Light Scattering: A Powerful Tool for In Situ Nanoparticle Sizing. Colloids Interfaces.

[87] Junghanns J.U.A.H., Müller R.H. (2008). Nanocrystal technology, drug delivery and clinical applications. Int. J. Nanomed..

[88] Müller R.H., Gohla S., Keck C.M. (2011). State of the art of nanocrystals—Special features, production, nanotoxicology aspects and intracellular delivery. Eur. J. Pharm. Biopharm..

[89] Malm A.V., Corbett J.C.W. (2019). Improved Dynamic Light Scattering using an adaptive and statistically driven time resolved treatment of correlation data. Sci. Rep..

[90] Stetefeld J., McKenna S.A., Patel T.R. (2016). Dynamic light scattering: A practical guide and applications in biomedical sciences. Biophys. Rev..

[91] Li C.L., Martini L.G., Ford J.L., Roberts M. (2005). The use of hypromellose in oral drug delivery. J. Pharm. Pharmacol..

[92] Maderuelo C., Zarzuelo A., Lanao J.M. (2011). Critical factors in the release of drugs from sustained release hydrophilic matrices. J. Control. Release.

[93] Siepmann J., Peppas N.A. (2012). Modeling of drug release from delivery systems based on HPMC. Adv. Drug Deliv. Rev..

[94] Wasilewska K., Winnicka K. (2019). Ethylcellulose—A Pharmaceutical excipient with multidirectional application in drug dosage forms development. Materials.

[95] Park C., Lee J.H., Jin G., Ngo H.V., Park J.-B., Tran T.T.D., Tran P.H.L., Lee B.-J. (2022). Release kinetics of hydroxypropyl methylcellulose governing drug release and hydrodynamic changes of matrix tablet. Curr. Drug Deliv..

[96] Vlad A., Pintea A., Pintea C., Rédai M., Antonoaea P., Bîrsan M., Ciurba A. (2025). Hydroxypropyl Methylcellulose—A Key Excipient in Pharmaceutical Drug Delivery Systems. Pharmaceutics.

[97] Colombo P., Bettini R., Santi P., Peppas N.A. (2000). Swellable matrices for controlled drug delivery: Gel-layer behaviour, mechanisms and optimal performance. Pharm. Sci. Technol. Today.

